# Impact of First-Line Antimicrobials on *Chlamydia trachomatis*-Induced Changes in Host Metabolism and Cytokine Production

**DOI:** 10.3389/fmicb.2021.676747

**Published:** 2021-08-13

**Authors:** Nadja Käding, Nis Schmidt, Celeste Scholz, Simon Graspeuntner, Jan Rupp, Kensuke Shima

**Affiliations:** ^1^Department of Infectious Diseases and Microbiology, University of Lübeck, Lübeck, Germany; ^2^German Center for Infection Research, Partner Site Hamburg-Lübeck-Borstel-Riems, Lübeck, Germany

**Keywords:** *Chlamydia trachomatis*, metabolism, doxycycline, azithromycin, cytokines

## Abstract

Urogenital infections with *Chlamydia trachomatis* (*C. trachomatis*) are the most common bacterial sexually transmitted diseases worldwide. As an obligate intracellular bacterium, chlamydial replication and pathogenesis depends on the host metabolic activity. First-line antimicrobials such as doxycycline (DOX) and azithromycin (AZM) have been recommended for the treatment of *C. trachomatis* infection. However, accumulating evidence suggests that treatment with AZM causes higher rates of treatment failure than DOX. Here, we show that an inferior efficacy of AZM compared to DOX is associated with the metabolic status of host cells. Chlamydial metabolism and infectious progeny of *C. trachomatis* were suppressed by therapeutic relevant serum concentrations of DOX or AZM. However, treatment with AZM could not suppress host cell metabolic pathways, such as glycolysis and mitochondrial oxidative phosphorylation, which are manipulated by *C. trachomatis*. The host cell metabolic activity was associated with a significant reactivation of *C. trachomatis* after removal of AZM treatment, but not after DOX treatment. Furthermore, AZM insufficiently attenuated interleukin (IL)-8 expression upon *C. trachomatis* infection and higher concentrations of AZM above therapeutic serum concentration were required for effective suppression of IL-8. Our data highlight that AZM is not as efficient as DOX to revert host metabolism in *C. trachomatis* infection. Furthermore, insufficient treatment with AZM failed to inhibit chlamydial reactivation as well as *C. trachomatis* induced cytokine responses. Its functional relevance and the impact on disease progression have to be further elucidated *in vivo*.

## Introduction

*Chlamydia trachomatis* (*C. trachomatis*) is an obligate intracellular bacterium causing genital tract infections. It is the most common bacterial sexually transmitted disease, with the highest prevalence in persons aged ≤24 years (National Center for HIV/AIDS, Viral Hepatitis, STD, and TB Prevention (U.S.). Division of STD Prevention, 2019). The WHO reported a total of 124.3 million cases of chlamydial infection worldwide, with around 1.8 million reported cases in the United States (National Center for HIV/AIDS, Viral Hepatitis, STD, and TB Prevention (U.S.). Division of STD Prevention, 2019; Rowley et al., 2019). An acute infection with *C. trachomatis* is often asymptomatic (World Health Organization, 2016), but recurrent infections, resulting in chronic chlamydial infection could be linked to inflammatory cytokines associated with pelvic inflammatory disease, ectopic pregnancy, and infertility (Brunham et al., 1985, 2015; Svensson et al., 1985; Reddy et al., 2004; Buchholz and Stephens, 2006; Mpiga et al., 2006; Hafner et al., 2008). Importantly, one in four women with chlamydial cervicitis has an asymptomatic pelvic inflammatory disease, and is more likely to become infertile (Wiesenfeld, 2017).

*Chlamydia trachomatis* is characterized by a unique development cycle with two distinct developmental forms: the infectious elementary bodies (EBs) and the replicating reticulate bodies (RBs; Fields and Hackstadt, 2002). EBs enter the host epithelial cell through endocytosis. After being taken up by the host cell, *C. trachomatis* creates an intracellular niche, so called inclusions in which EBs differentiate into RBs. During its developmental cycle, *C. trachomatis* manipulates host cell metabolism for its growth due to chlamydial truncated metabolic pathways (Stephens et al., 1998; Omsland et al., 2012). Hexokinase catabolizes the conversion of glucose to glucose-6-phosphate in glycolysis. The hexokinase gene, however, is lacking in the chlamydial genome (Omsland et al., 2014). Therefore, host glucose metabolism is essential for *C. trachomatis* to sustain its own glycolytic activity. Furthermore, mitochondria are known as power plants of the host cell, as their respiration efficiently generates most of the ATP under oxygenic conditions in normal healthy cells (Yetkin-Arik et al., 2019). *Chlamydia trachomatis* not only acquires host cell-derived ATP, but also it has to hijack mitochondria-derived metabolites for its replication due to the truncated chlamydial tricarboxylic acid (TCA) cycle (Stephens et al., 1998; Kubo and Stephens, 2001; Rajeeve et al., 2020; Shima et al., 2021).

Importantly, cellular metabolic activity and host immune responses are highly linked (Buck et al., 2017; Kouidhi et al., 2017). It is known that immune cells drastically change their metabolic activity to cope with antigens, resulting in production of large amounts of effector molecules including cytokines (Loftus and Finlay, 2016). Thus, various proinflammatory cytokines including IL-8 and IL-6, which increase inflammation, are secreted from *C. trachomatis* infected immune cells as well as cervical epithelial cells (Rasmussen et al., 1997; Reddy et al., 2004; Mpiga et al., 2006).

Antimicrobials are used for treatment of bacterial infection as well as reduction of the host immune response (Ankomah and Levin, 2014). First-line antimicrobials such as doxycycline (DOX) or azithromycin (AZM) are recommended for the treatment of *C. trachomatis* infection (Geisler et al., 2015). While both antimicrobials are effective to eradicate *C. trachomatis* (Geisler et al., 2015), subinhibitory concentrations of these drugs could induce chlamydial persistence known as a viable but noncultivable state (Dreses-Werringloer et al., 2001; Gieffers et al., 2004; Xue et al., 2017). Furthermore, accumulating evidence suggests that treatment failure occurs more often during AZM treatment than with DOX (Batteiger et al., 2010; Geisler et al., 2015; Dukers-Muijrers et al., 2019). Indeed, AZM causes up to 14% of treatment failure in female urogenital chlamydial infections (Batteiger et al., 2010; Geisler et al., 2015; Dukers-Muijrers et al., 2019).

While the efficacy of AZM is inferior to DOX in *C. trachomatis* infection (Batteiger et al., 2010; Geisler et al., 2015; Dukers-Muijrers et al., 2019), little is known about the metabolic status of *C. trachomatis* and host cells under treatment. Therefore, our study aims to determine the impact of first-line antimicrobials on host-pathogen metabolism as well as cytokine production.

## Materials and Methods

### Chemicals

All chemicals were purchased from Sigma Aldrich (Deisenhofen, Germany).

### Cell Culture and Infection With *C. trachomatis*

A total of 2.5 × 10^5^ HeLa-229 cells (ATCC CCL-2.1) were seeded per well in six-well plates (Greiner Bio-One GmbH, Frickenhausen, Germany) with RPMI1640 medium (Invitrogen GmbH, Darmstadt, Germany) supplemented with 5% fetal bovine serum (FBS; Gibco/Invitrogen, Karlsruhe, Germany). Cells were cultured for 24 h under 5% CO_2_ at 37°C. Afterward, the cells were infected with *C. trachomatis* serovar L2 at the multiplicity of infection (MOI) of 1.4.

### Recovery Assay

At 20 hours post infection (hpi), *C. trachomatis* infected cells were treated with DOX (2 μg/ml; Siewert et al., 2005) or AZM (0.5 or 5 μg/ml; Cooper et al., 1990) for 24 h. After washing, *C. trachomatis* infected cells were detached with a cell scraper and resuspended in fresh growth medium. The cell suspension was disrupted with glass beads for 10 min on a vibrating shaker to release *C. trachomatis* from host cells. Serial dilutions of the suspension were inoculated in confluent HEp-2 cell monolayers with 1 μg/ml cycloheximide. The plate was further centrifuged at 700 × *g* for 1 h at 35°C and incubated for 36 h. Subsequently, samples were fixed by methanol and visualized by FITC-labeled monoclonal chlamydial-LPS antibodies (Dako, Hamburg, Germany). Recoverable *C. trachomatis* were calculated as infection forming units (IFUs)/μl by observation of 10 microscopy fields (40×magnification) using a fluorescence microscope (Axiovert 25, Zeiss, Göttingen, Germany) and a LD Achroplan 40x/0.60 Korr objective (Zeiss).

### Reactivation Assay

Reactivation assay was performed as described previously with minor modifications (Belland et al., 2003; Shima et al., 2021). At 20 hpi, *C. trachomatis* infected cells were treated with 2 μg/ml of DOX, 0.5 and 5 μg/ml of AZM for 24 h (Supplementary Figure S1). The cells were washed with RPMI 1640 medium twice to remove the remaining antimicrobials and further cultured in the presence or absence of 3 mM of 2-deoxyglucose (2-DG) or 0.2 μM of antimycin A for 24 h. Reactivated *C. trachomatis* was determined as described in a recovery assay.

### Metabolic Assay

The Seahorse XF24 Analyzer (Agilent Technologies, California, United States) was used to measure the oxygen consumption rate (OCR) and the extracellular acidification rate (ECAR) under the antimicrobial treatment. A total of 1.5 × 10^4^ HeLa cells were seeded and cultured in the XF24 cell culture microplate (Agilent Technologies). At 20 hpi *C. trachomatis* infected cells were treated with DOX (2 µg/ml) or AZM (0.5 or 5 µg/ml) for 24 h. The XF Cell Mito Stress Test Kit or the Glycolysis Stress Test Kit was used following Agilent Technologies manufacturer’s instructions with chemical concentrations [Mito Stress Test: oligomycin (0.5 μM), FCCP (0.2 μM) and antimycin A (1 μM) plus rotenone (1 μM); Glycolysis Stress Test: glucose (10 mM), oligomycin (0.5 μM), and 2-DG (100 μM)].

### FLIM of [τ_2_-NAD(P)H] by Two-Photon Microscopy

A total of 5 × 10^5^ HeLa cells were seeded on 40 mm coverslips. At 20 hpi *C. trachomatis* infected cells were treated with DOX (2 µg/ml) or AZM (0.5 or 5 µg/ml) for 24 h. Shortly before imaging, the coverslip was set up in the MiniCeM chamber (Jenlab). Fluorescence lifetime imaging (FLIM) of protein-bound NAD(P)H [τ_2_-NAD(P)H] was performed with a two-photon laser scanning microscope, the DermaInspect (JenLab). A tunable infrared titanium-sapphire femtosecond-laser (710–920 nm tuning range; MaiTai; Spectra Physics, Darmstadt, Germany) was used as an excitation source at 730 nm excitation for FLIM of NAD(P)H. For the visualization, we used a 40 x/1.3 Plan-Apochromat oil-immersion objective. Residual excitation light was blocked by a blue emission filter (BG39, Schott AG). FLIM data were collected by a time-correlated single-photon counting (TCSPC) system (PMH-100-0, SPC-830, Becker & Hickl, Berlin, Germany). Single photon counting was done for 49.7 s per image. Afterward, FLIM data were analyzed by SPCImage software (Version 5.0; Becker & Hickl GmbH, Berlin Germany). In every image, three cells were analyzed from 10 visual fields per chamber on three independent measurements. The visual field was 110 × 110 μm^2^ corresponding to 256 × 256 pixels. Lifetime decay curves were fitted to a double exponential decay model. The instrument response function (IRF), which was included in the fit model, was measured from the second harmonic generation signal of beta-barium-borate crystal. For image analysis, a region of interest (ROI) was selected within the chlamydial inclusion.

### Analysis of Cytokine Expression

A total of 2.5 × 10^5^ HeLa cells were seeded per well in six-well plates. At 20 hpi *C. trachomatis* infected cells were treated with DOX (2 µg/ml) or AZM (0.5 or 5 µg/ml) for 24 h. Total RNA was isolated after 44 hpi using the NucleoSpin RNA II kit (Macherey-Nagel) and reverse-transcribed into cDNA (RevertAid First Strand cDNA Synthesis kit, Thermo Fischer Scientific). PCR amplification was performed by the LightCycler Detection System. Relative quantification of IL-6 (forward CCTTCCAAAGATGGCTGAAA, reverse CAGGGGTGGTTATTGCATCT) or IL-8 (forward CCAGGAAGAAACCACCGGA, reverse GAAATCAGGAAGGCTGCCAAG) mRNA expression levels were normalized by the endogenous control β-actin gene (forward: GCCAACCGCGAGAAGATGA reverse: CATCACGATGCCAGTGGTA) using the threshold cycle (2^ΔΔCT^) method (Livak and Schmittgen, 2001).

### Impact of Metabolic Inhibitors on Cytokine Expression

A total of 2.5 × 10^5^ HeLa cells were seeded per well in six-well plates. Each antimicrobial and inhibitor such as 2 μg/ml of DOX, 0.5 and 5 μg/ml of AZM, 3 mM of 2-DG, or 0.2 μM of antimycin A was added at the time of infection and cultured for 24 h. The analysis of cytokine expression in the previous method section was applied for this assay.

### Microbial Composition in the Human Vagina

Analysis of microbial composition of the human vagina has been undertaken using 16S amplicon sequencing of the V1/V2 hypervariable region as described elsewhere (Graspeuntner et al., 2018). Visualization of the microbial composition was performed using R version 3.6.3 (R Core Team, 2020).^1^ Data are taken from a study approved by the ethics committee of the University of Lübeck (reference number 11–185).

### Statistics

Data are indicated as mean ± SEM. Statistical analysis was performed by GraphPad Prism 7 statistical software. When three or more groups were compared in the experiment, Sidak’s multiple comparisons were used in cases that ANOVA showed statistical significance (values of *p* ≤ 0.05). Data between two groups were evaluated using the Student’s *t*-test. In Sidak’s multiple comparison and the Student’s *t*-test, values of *p* ≤ 0.05 were considered as statistically significant.

## Results

### The Efficacy of a Therapeutic Relevant Serum Concentration of DOX and AZM Against *C. trachomatis*

In our previous study, we confirmed that therapeutic relevant serum concentrations of either DOX (2 μg/ml) or AZM (0.5 μg/ml) could completely block *C. trachomatis* infection when antimicrobials were added right after infection (Shima et al., 2011). However, *C. trachomatis* infections are generally established before initiation of antimicrobial therapy. Therefore, *C. trachomatis* infected cells were treated with a therapeutic relevant serum concentration of DOX or AZM 20 hpi when chlamydial inclusions could already be observed microscopically in this study.

**Figure 1:**
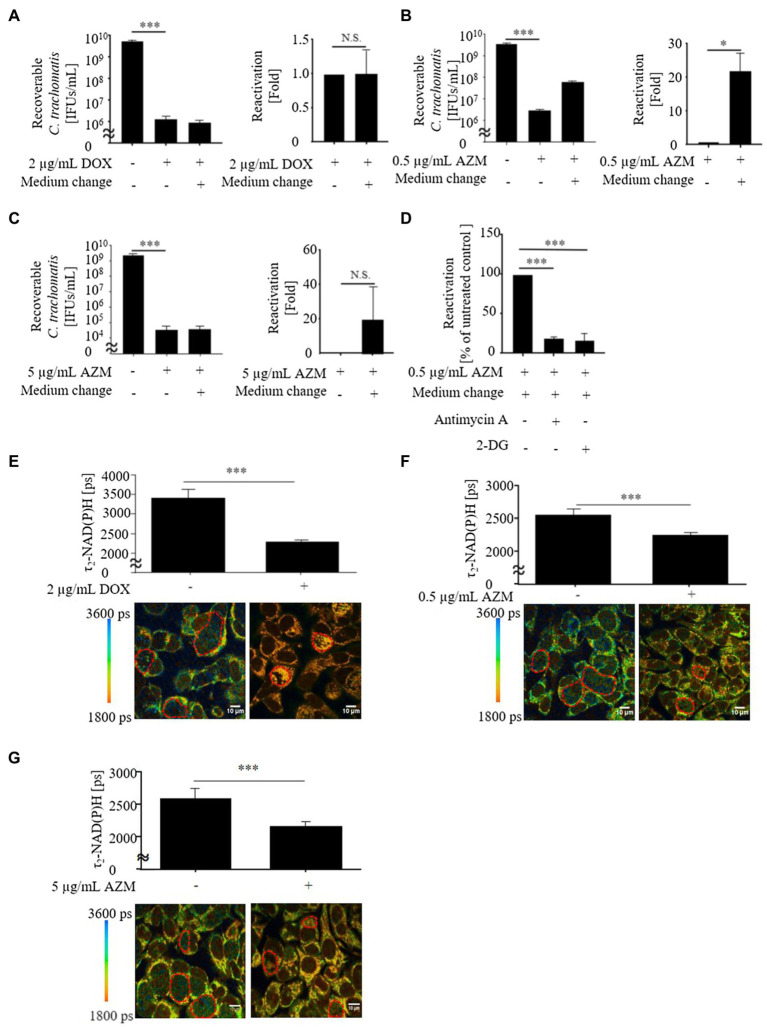
Viability and metabolic activity of *Chlamydia trachomatis* in HeLa cells after treatment with 2 μg/ml of doxycycline (DOX), 0.5 or 5 μg/ml of azithromycin (AZM). **(A)** Recoverable *C. trachomatis* after treatment with 2 μg/ml of DOX for 24 h and reactivation of *C. trachomatis* after medium change. **(B)** Recoverable *C. trachomatis* after treatment with 0.5 μg/ml of AZM for 24 h and reactivation of *C. trachomatis* after medium change. **(C)** Recoverable *C. trachomatis* after treatment with 5 μg/ml of AZM for 24 h and reactivation of *C. trachomatis* after medium change. **(D)** Reactivation with 3 mM of 2-DG or 0.2 μM of antimycin A. **(E)** Upper panel: Quantitative analysis of τ_2_-NAD(P)H signals in the chlamydial inclusions. Bottom panel: Color-coded images of τ_2_-NAD(P)H in chlamydial inclusions in the presence or absence of 2 μg/ml of DOX 44 hours post infection (hpi). **(F)** Upper panel: Quantitative analysis of τ_2_-NAD(P)H signals in the chlamydial inclusions. Bottom panel: Color-coded images of τ_2_-NAD(P)H in chlamydial inclusions in the presence or absence of 0.5 μg/ml of AZM 44 hpi. **(G)** Upper panel: Quantitative analysis of τ_2_-NAD(P)H signals in the chlamydial inclusions. Bottom panel: Color-coded images of τ_2_-NAD(P)H in chlamydial inclusions in the presence or absence of 5 μg/ml of AZM 44 hpi. *Ct*, *C. trachomatis*; DOX, doxycycline; AZM, azithromycin; 2-DG, 2-deoxyglucose (**A-D**: *n* = 3, **E-G**: ROIs; Mean ± SEM, Student *t*-Test and Sidak’s multiple comparison: ^*^p ≤ 0.05; ^***^p ≤ 0.001; and N.S., not significant).

To determine the production of chlamydial infectious progeny after treatment with DOX and AZM, we performed a recovery assay. In this assay, we observed that DOX and AZM efficiently blocked production of infectious progeny (Figures 1A,B).

In previous studies, we demonstrated that FLIM of protein-bound NAD(P)H [τ_2_-NAD(P)H] in the inclusion can be used as an indicator for intracellular chlamydial metabolic activity (Szaszák et al., 2011; Käding et al., 2017; Shima et al., 2018, 2021). Therefore, we further analyzed τ_2_-NAD(P)H in chlamydial inclusions during treatment with DOX and AZM (Figures 1E,F). After 24 h of antimicrobial treatment, either 2 μg/ml of DOX or 0.5 μg/ml of AZM led to a decrease of τ_2_-NAD(P)H in the chlamydial inclusion, indicating reduced metabolic activity of *C. trachomatis* (Figures 1E,F).

### Impact of DOX and AZM on Metabolism in *C. trachomatis* Infected Cells

*Chlamydia trachomatis* manipulates host glycolytic and mitochondrial activities to acquire host cell-derived metabolites for its intracellular replication (Shima et al., 2018, 2021; Maffei et al., 2020; Rajeeve et al., 2020). Therefore, we analyzed whether the treatment with 2 μg/ml of DOX or 0.5 μg/ml of AZM could restore the manipulated host cell metabolism.

**Figure 2:**
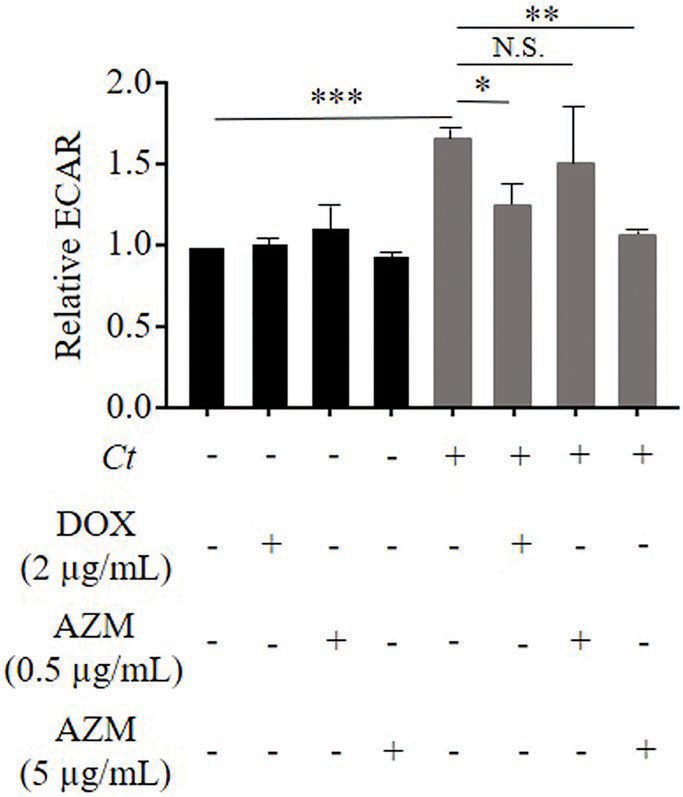
Impact of DOX and AZM on glycolytic activity in *C. trachomatis* infected HeLa cells. Cells were treated with 2 μg/ml of DOX, 0.5 or 5 μg/ml of AZM for 24 h. Glycolysis was measured by Glycolysis stress test kit. ECAR, extracellular acidification rate; *Ct*, *C. trachomatis*; DOX, doxycycline; AZM, azithromycin (*n* = 3–9; Mean ± SEM, Sidak’s multiple comparison: ^*^p ≤ 0.05; ^**^p ≤ 0.01; ^***^p ≤ 0.001; and N.S. not significant).

As expected, *C. trachomatis* significantly increased glycolysis determined by the ECAR in *C. trachomatis* infected cells compared to uninfected cells (Figure 2). Glycolysis in *C. trachomatis* infected cells was reduced during treatment with DOX compared to *C. trachomatis* infected cells (Figure 2). In contrast, treatment with 0.5 μg/ml of AZM could not restore glycolysis to the baseline level in *C. trachomatis* infected cells (Figure 2).

**Figure 3:**
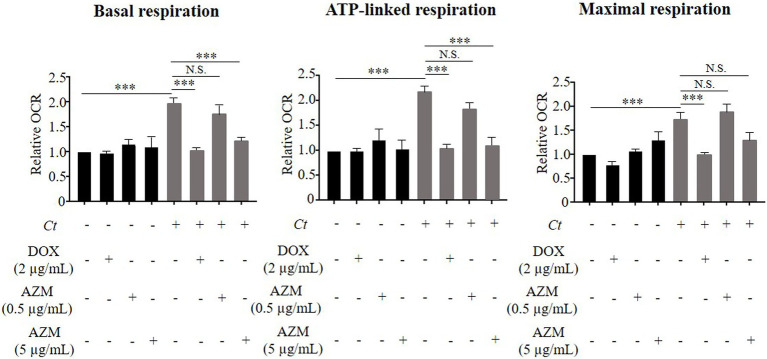
Impact of DOX and AZM on mitochondrial activity in *C. trachomatis* infected HeLa cells. Cells were treated with 2 μg/ml of DOX, 0.5 or 5 μg/ml of AZM for 24 h. Mitochondrial activity, indicated by basal respiration, ATP-linked respiration and maximal respiration was measured by Mito Stress test kit. OCR, oxygen consumption rate; *Ct*, *C. trachomatis*; DOX, doxycycline; AZM, azithromycin (*n* = 3–12; Mean ± SEM, Sidak’s multiple comparison: ^***^p ≤ 0.001; N.S. not significant).

We observed a similar trend in mitochondrial activity, determined by the cellular OCR. *Chlamydia trachomatis* enhanced mitochondrial activity as shown by a significant induction of basal respiration, ATP-linked respiration and maximal respiration (Figure 3). This effect was restored close to the baseline level by treatment with 2 μg/ml of DOX (Figure 3), but not by 0.5 μg/ml of AZM (Figure 3).

We therefore increased the concentration of AZM to 5 μg/ml to check for dose-dependent effects on host cell metabolism. This concentration of AZM reduced the chlamydial progeny and τ_2_-NAD(P)H in chlamydial inclusions after 24 h treatment (Figures 1C,G). Furthermore, upregulated glycolysis and mitochondrial activity shown by basal respiration and ATP-linked respiration in *C. trachomatis* infection were also restored close to the baseline level by treatment with 5 μg/ml of AZM (Figures 2, 3).

### Reactivation of *C. trachomatis* After Treatment With DOX and AZM

Although production of infectious progeny was blocked by 0.5 μg/ml of AZM (Figure 1B), maintained glycolysis and mitochondrial activity indicates that some *Chlamydia* may still survive as a noncultivable state during treatment with 0.5 μg/ml of AZM. Since this state of *Chlamydia* can be reactivated after the removal of antimicrobials (Gieffers et al., 2004; Xue et al., 2017), we performed a reactivation assay after antimicrobial treatment (Supplementary Figure S1). While reactivation of *C. trachomatis* was not observed under treatment with 2 μg/ml of DOX (Figure 1A), significant amounts of *C. trachomatis* were reactivated after treatment with 0.5 μg/ml of AZM (Figure 1B). Furthermore, no reactivation of *C. trachomatis* was detected when host cell metabolism was restored close to the baseline level by 5 μg/ml of AZM (Figure 1C). To check whether *C. trachomatis* enhanced glycolysis and mitochondrial activity are linked to reactivation of *C. trachomatis* after antimicrobial treatment, we blocked glycolysis and mitochondrial activity using glycolytic inhibitor, 2-DG and mitochondrial complex III inhibitor, antimycin A during reactivation. Reactivated *C. trachomatis* was reduced when cells were treated with either 2-DG or antimycin A compared to non-treated control cells (Figure 1D).

### Impact of DOX and AZM on Cytokine Production in *C. trachomatis* Infected Cells

The analysis of host cell metabolism and reactivation assays revealed that host-pathogen interaction is not completely blocked by treatment with 0.5 μg/ml of AZM. Therefore, *C. trachomatis* might elicit a host inflammatory response even under treatment with AZM. To elucidate this, we analyzed cytokine expression in *C. trachomatis* infected cells under treatment with DOX and AZM.

**Figure 4:**
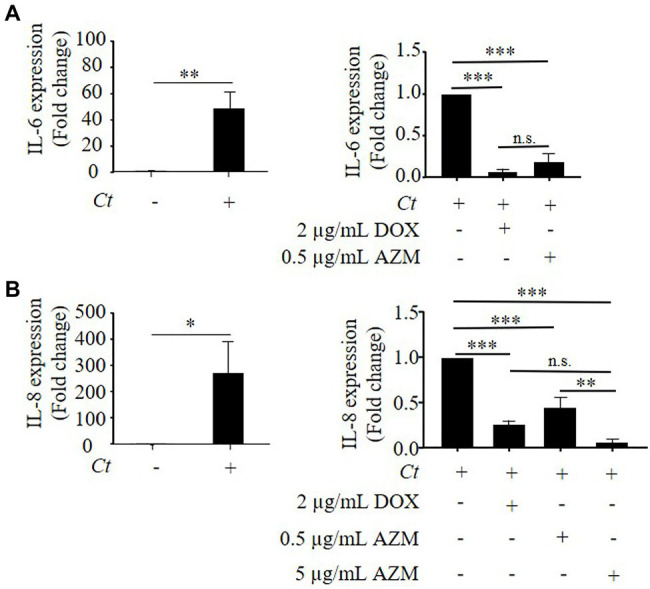
Expression of IL-8 and IL-6 in *C. trachomatis* infected HeLa cells under treatment with DOX and AZM. **(A)** Quantitative analysis of IL-6 mRNA expression in *C. trachomatis* infected HeLa cells under treatment with 2 μg/ml of DOX or 0.5 μg/ml of AZM for 24 h. **(B)** Quantitative analysis of IL-8 mRNA expression in *C. trachomatis* infected HeLa cells under treatment with 2 μg/ml of DOX or 0.5 or 5 μg/ml of AZM for 24 h. *Ct*, *C. trachomatis*; DOX, doxycycline; AZM, azithromycin (*n* = 3; Mean ± SEM, Student’s *t*-Test and Sidak’s multiple comparison: ^*^p ≤ 0.05; ^**^p ≤ 0.01; ^***^p ≤ 0.001; and N.S. not significant).

As it was shown in previous studies (Buchholz and Stephens, 2006; Cunningham et al., 2013), we observed that *C. trachomatis* led to increased expression of IL-8 and IL-6 (Figures 4A,B). In line with the inhibitory effect of DOX on reactivation assays, treatment with 2 μg/ml of DOX reduced IL-8 and IL-6 expression by 92 ± 3 and 73 ± 2%, respectively compared to non-treated controls (Figures 4A,B). Treatment with 0.5 μg/ml of AZM reduced IL-6 expression by 81 ± 9% (Figure 4A), while IL-8 expression was only attenuated by 40 ± 10% compared to non-treated conditions (Figure 4B). Applying the higher AZM dose of 5 μg/ml that restored host cell metabolism close to the baseline level, IL-8 expression levels were further reduced by 92 ± 4% compared to non-treated conditions (Figure 4B).

To elucidate the link between *C. trachomatis* disturbed host cell metabolism and cytokine expression, we analyzed IL-8 expression in *C. trachomatis* infection under treatment with 2-DG or antimycin A. As a result, IL-8 expression was increased in *C. trachomatis* infection and this effect was further enhanced when mitochondrial functions were impaired by antimycin A (Supplementary Figure S2). This indicates that disturbed host cell metabolic activity has an impact on cytokine expression in *C. trachomatis* infection.

## Discussion

Doxycycline and AZM are the two most commonly prescribed antimicrobials for uncomplicated urogenital tract infections with *C. trachomatis* (World Health Organization, 2016). However, treatment failure resulting in recurrence of *C. trachomatis* after antimicrobial therapy is a considerable issue (Batteiger et al., 2010; Geisler et al., 2015; Dukers-Muijrers et al., 2019). Either 2 μg/ml of DOX or 5 μg/ml of AZM inhibits *C. trachomatis* induced host metabolic activation as well as production of progeny, whereas *C. trachomatis* still survived and hence activated host metabolism under treatment with therapeutic serum concentration of AZM. Since *C. trachomatis* fundamentally needs host cell metabolites for its replication, it manipulates host cell glycolysis and mitochondria (Chowdhury et al., 2017; Shima et al., 2018, 2021; Kurihara et al., 2019; Ende and Derré, 2020; Maffei et al., 2020; Rajeeve et al., 2020). Therefore, we suggest that the suppression of host metabolism enhanced by *C. trachomatis* is also an important factor for treatment of *C. trachomatis* infections.

To elucidate treatment failure caused by AZM, several groups investigated various factors such as the presence of resistance genes and the pharmacokinetics (Vodstrcil et al., 2017; Shao et al., 2020). Vodstrcil et al. (2017) demonstrated that 1 g of AZM can reach the genital tract and maintains adequate concentrations in the vagina. On the other hand, although, it is known that AZM is extensively distributed into tissues, concentrations vary in different host compartments (Matzneller et al., 2013). For example, concentrations of AZM in soft tissues such as the extracellular space of muscle and adipose tissues that can be found in a stromal constituent of the uterine cervix, were lower than serum concentration (Doldan et al., 2009; Matzneller et al., 2013; El Hadi et al., 2018). This indicates that *C. trachomatis* infection in these areas cannot be eradicated by serum concentration of AZM. Furthermore, microenvironmental conditions such as the local pH and a low oxygen concentration also reduce the efficacy of antimicrobials (Carbon, 1998; Juul et al., 2007; Shima et al., 2011). *Lactobacilli* are a dominant bacterial community in human vaginal microbiota (Supplementary Figure S3) and maintain the vaginal pH between 4.2 and 5.0 (Ravel et al., 2011). While a pKa of AZM is 8.5 and a pH of 8 is the optimal pH for an active form of AZM, the efficacy of macrolides reduces at a pH lower than 6 (Carbon, 1998; Kong et al., 2017). Importantly, a pKa of DOX is 3.09 and it is effective under low pH conditions (Bogardus and Blackwood, 1979; Smith et al., 2019). Besides the uterine cervix and microenvironmental factors, metabolic and inflammatory effects have to be considered as well.

As observed in our study, therapeutic serum concentration of AZM, could not efficiently suppress cytokine expression in *C. trachomatis* infection. Importantly, the link between host metabolic activity and immune responses has been demonstrated in various studies (Buck et al., 2017; Kouidhi et al., 2017). Mitochondria-derived reactive oxygen species (mtROS) are generated in the mitochondrial electron transport chain (ETC; Quinlan et al., 2011). Several groups demonstrated that inhibition of mtROS could block activation of mitogen-activated protein kinase (MAPK) and production of cytokines in different cells such as human derived peripheral blood mononuclear cells (PBMCs) and mouse embryonic fibroblasts (MEFs; Kamata et al., 2005; Bulua et al., 2011). This indicates that mtROS is relevant for the induction of host cell inflammatory responses. Importantly, we and another group demonstrated that chlamydiae can induce ROS in epithelial cells at 24 hpi (Boncompain et al., 2010; Käding et al., 2017). Furthermore, it is known that ETC complex III generates superoxide at high rates in the presence of antimycin A (Quinlan et al., 2011). Accordingly, we showed that increased IL-8 expression by *C. trachomatis* is further enhanced in antimycin A impaired mitochondrial functions. Therefore, we suggest that the therapeutic serum concentration of AZM cannot restore disturbed mitochondrial activity to physiological conditions, resulting in insufficient suppression of IL-8 expression in *C. trachomatis* infected epithelial cells. IL-8 induces migration of neutrophils that are a first-line defense in the immune response (Gainet et al., 1998; Akkoyunlu et al., 2001; Martínez-García et al., 2015; Lehr et al., 2018). Although neutrophils play a key role to eradicate pathogens, they also cause severe inflammation in chronic infections (Gainet et al., 1998; Akkoyunlu et al., 2001; Martínez-García et al., 2015; Lehr et al., 2018).

Taken together, efficacy of AZM is inferior to DOX and this might account for the higher rate of treatment failure in AZM therapy. Our findings highlight that treatment with the therapeutic relative serum concentration of AZM fails to restore disturbed host cell metabolism to the physiological conditions, leading to insufficient reduction of pro-inflammatory cytokine responses in *C. trachomatis* infections.

## Data Availability Statement

The original contributions presented in the study are included in the article/Supplementary Material, further inquiries can be directed to the corresponding author.

## Ethics Statement

The studies involving human participants were reviewed and approved by the Ethics Committee of the University of Lübeck (reference number 11–185). The patients/participants provided their written informed consent to participate in this study.

## Author Contributions

NK, NS, CS, JR, and KS wrote the manuscript. NK, JR, and KS designed this study. NK, NS, CS, SG, and KS performed biological assays. All authors contributed to the article and approved the submitted version.

## Conflict of Interest

The authors declare that the research was conducted in the absence of any commercial or financial relationships that could be construed as a potential conflict of interest.

## Publisher’s Note

All claims expressed in this article are solely those of the authors and do not necessarily represent those of their affiliated organizations, or those of the publisher, the editors and the reviewers. Any product that may be evaluated in this article, or claim that may be made by its manufacturer, is not guaranteed or endorsed by the publisher.

## Footnotes

^1^ https://www.R-project.org/
