# Supplementary material for: Impact of First-Line Antimicrobials on Chlamydia trachomatis-Induced Changes in Host Metabolism and Cytokine Production
Source: Front Microbiol. 2021 Aug 13;12:676747. doi: 10.3389/fmicb.2021.676747 (PMC8414654; doi:10.3389/fmicb.2021.676747)
Supplement: Supplementary file 1 [file Data_Sheet_1.docx]

Supplementary Material


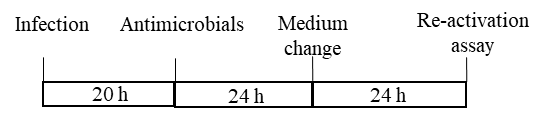


**Supplementary Figure 1.** Representation of the experimental setting of reactivation assay. HeLa cells were infected with *C. trachomatis* for 20 h and afterward treated with 2 µg/mL of DOX or 0.5 or 5 µg/mL of AZM for 24 h. At 44 hpi, the medium was changed and further cultured in the presence or absence of 3 mM 2-DG or 0.2 µM antimycin A for 24 h.


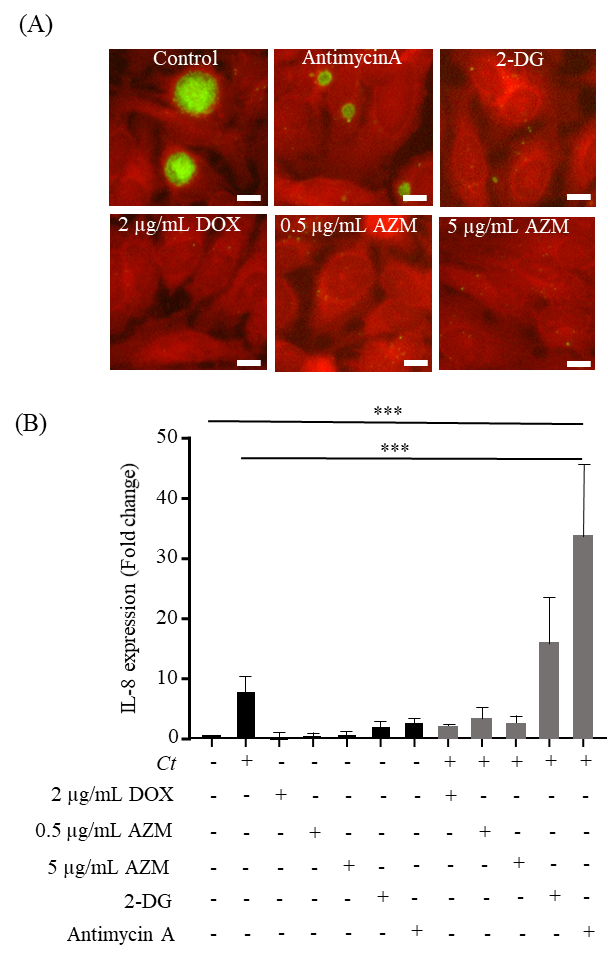


**Supplementary Figure 2:** Expression of IL-8 in *C. trachomatis* infected host cells during treatment with antimicrobials and metabolic inhibitors. (A) Representative images of chlamydial inclusions in 2 µg/mL DOX, 0.5 or 5 µg/mL AZM, 3 mM of 2-DG or 0.2 µM of antimycin A-treated and nontreated cells at 24 hpi. Bars = 10 µm. (B) Quantitative analysis of IL-8 mRNA expression in *C. trachomatis* infected cells with 2 µg/mL DOX, 0.5 or 5 µg/mL AZM, 3 mM of 2-DG or 0.2 µM of antimycin A. (n=3; Mean ± SEM; Sidak´s multiple comparison: ***, *p* ≤ 0.001).

**
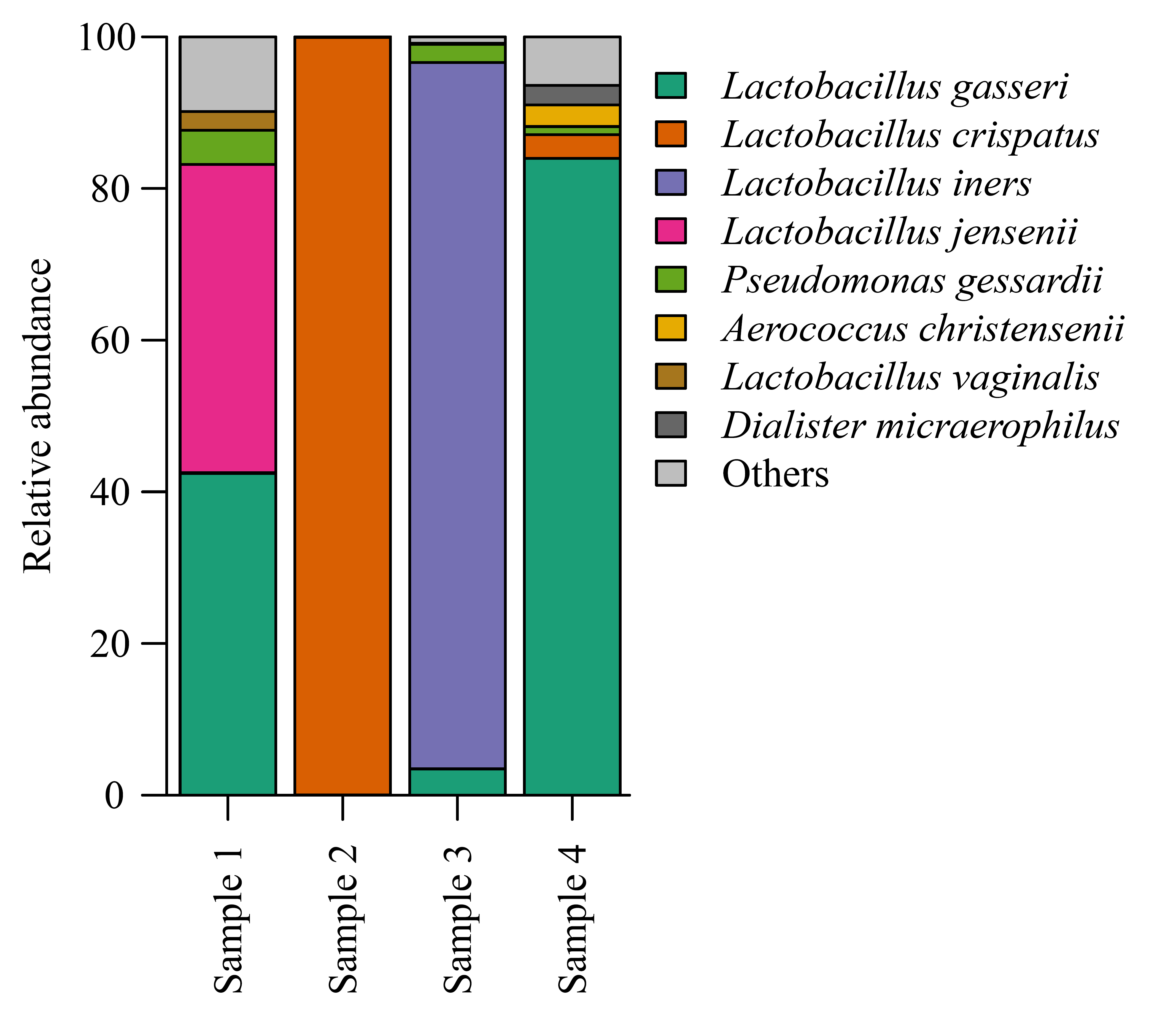
**

**Supplementary Figure 3:** Lactobacillus dominated bacterial communities in the human vagina. Different representative *Lactobacillus*-dominated bacterial communities as shown by 16S-based microbiota assessment.
